# Impact of Phosphate, Potassium, Yeast Extract, and Trace Metals on Chitosan and Metabolite Production by *Mucor indicus*

**DOI:** 10.3390/ijms17091429

**Published:** 2016-08-30

**Authors:** Zahra Safaei, Keikhosro Karimi, Akram Zamani

**Affiliations:** 1 Department of Chemical Engineering, Isfahan University of Technology, Isfahan 84156-83111, Iran; zahra.safaie@gmail.com (Z.S.); karimi@cc.iut.ac.ir (K.K.); 2 Swedish Centre for Resource Recovery, University of Borås, Borås 50190, Sweden

**Keywords:** chitosan, ethanol, *Mucor indicus*, phosphate, potassium, trace metals, yeast extract

## Abstract

In this study the effects of phosphate, potassium, yeast extract, and trace metals on the growth of *Mucor indicus* and chitosan, chitin, and metabolite production by the fungus were investigated. Maximum yield of chitosan (0.32 g/g cell wall) was obtained in a phosphate-free medium. Reversely, cell growth and ethanol formation by the fungus were positively affected in the presence of phosphate. In a phosphate-free medium, the highest chitosan content (0.42 g/g cell wall) and cell growth (0.66 g/g sugar) were obtained at 2.5 g/L of KOH. Potassium concentration had no significant effect on ethanol and glycerol yields. The presence of trace metals significantly increased the chitosan yield at an optimal phosphate and potassium concentration (0.50 g/g cell wall). By contrast, production of ethanol by the fungus was negatively affected (0.33 g/g sugars). A remarkable increase in chitin and decrease in chitosan were observed in the absence of yeast extract and concentrations lower than 2 g/L. The maximum chitosan yield of 51% cell wall was obtained at 5 g/L of yeast extract when the medium contained no phosphate, 2.5 g/L KOH, and 1 mL/L trace metal solution.

## 1. Introduction

Discovered some hundreds of years ago, *Mucor indicus* (*M. indicus*) is one of the most well-known strains of zygomycetes fungi. The importance of this fungus is not only related to its application in traditional foods but also to its superior ability in the production of ethanol and chitosan [1].

The inability of *Saccaromyces cerevisiae* (*S. cerevisiae*) to consume the pentoses available in lignocellulosic hydrolysates is among the challenges in the development of a second generation of bioethanol. Replacement of *S. cerevisiae* with *M. indicus* is one of the suggested solutions to this problem. The fungus consumes both hexoses and pentoses and produces ethanol with yields comparable to those achieved from *S. cerevisiae* [1,2]. Chitosan is another valuable co-product obtained during fermentation of the fungus. This cationic biopolymer, which is stored in the cell wall of zygomycetes fungi, has shown the potential for replacement of shellfish chitosan [1,3,4]. Recent studies indicate the impact of composition of culture medium on chitosan and ethanol production. Javarska et al. [5] showed that the production of chitosan by *Absidia orchidis* can be regulated in the presence of iron and manganese ions, while cobalt ions prevented the growth of fungi. Goksungur [6] showed a 15% increase in chitosan production by *Rhizopus oryzae* fermentation by optimizing the initial glucose concentration, aeration rate, and agitation speed. Yoshyhara et al. [7] reported that adding d-psicosein to a medium including a small amount of d-glucose increases the production of chitosan by *Rhizopus oryzae* (*R. oryzae*) YPF-61. Sues et al. [8] investigated an optimal medium for ethanol production by *M. indicus* and reported a significant increase in biomass, ethanol, and glycerol yield by adding 2.5 g/L yeast extract to a basal medium. Chatterjee et al. [9] investigated the influence of gibberellic acid, auxins, and kinetin plant growth hormones on growth and production of chitosan by *R. oryzae* in a whey medium. The effect of several nitrogen sources was studied by Jiangand et al. [10], who showed that soy extract increases the production of chitosan. Mohammadi et al. [11] reported that the filamentous form of the fungus as well as the absence of potassium hydrogen phosphate favors chitosan production. Recently, we have demonstrated the impact of plant growth hormones on ethanol and chitosan production by *M. indicus* [12].

Although production of ethanol and chitosan from *M. indicus* has been the subject of several studies, much more investigation is needed to be able to fully understand the influence of different growing conditions on the fungus growth and formation of chitosan and ethanol by this fungus.

The objective of this study was to investigate the effect of the concentration of different components in a semi-synthetic culture medium, which is usually used in ethanolic fermentations of *M. indicus*, on fungal growth and the production of different products. Potassium hydrogen phosphate was replaced with phosphoric acid and potassium hydroxide and the effects of potassium and phosphate were studied individually. Furthermore, the culture media was supplemented with trace metals and changes in product formation were investigated. Finally, at optimal levels of the studied parameters, for chitosan production, the effect of yeast extract on cell growth and formation of different products was examined.

## 2. Results

The zygomycetes fungus *M. indicus* was cultivated on different concentrations of phosphate, potassium, trace metals, and yeast extract and the changes in cell growth, chitosan formation, and ethanol production were investigated.

### 2.1. The Effects of Phosphate

#### 2.1.1. Effects of Phosphate on Biomass Production and Its Composition

To study the effect of phosphate, the concentrations of KOH, yeast extract, and trace metals were kept constant (at 1.44, 5.00, and 0.00 g/L, respectively). According to Table 1, an increase in biomass yield was observed at increasing concentrations of phosphoric acid. By adding 0.25 g/L of H_3_PO_4_, a 6% increase in biomass yield was obtained compared to the medium with no phosphate. This continued to increase with an increase in the phosphoric acid concentration. Although the presence of phosphate had a positive effect on the cell growth, no significant change in the protein content of the biomass was observed at various phosphate levels (Table 1). Alkali-insoluble material (AIM) yield was 17%–21% of biomass and generally higher yields were observed at lower phosphate levels (Table 1).

#### 2.1.2. Effects of Phosphate on Ethanol and Glycerol Production

Ethanol and glycerol were formed as the major metabolites of the fungus at all concentrations of phosphate. A maximum of 12.7% difference was observed in ethanol yields at different phosphate levels. The highest and lowest ethanol yields were 0.47 and 0.41 g/g sugar, which were achieved in cultures with 0.5 and 0.0 g/L phosphoric acid, respectively. Glycerol yield (0.06–0.09 g/g sugar) was also not significantly affected by the concentration of phosphate (Table 1).

#### 2.1.3. Effects of Phosphate on Cell Wall Composition

Glucosamine, *N*-acetyl glucosamine (as main precursors of chitosan and chitin, respectively), and phosphates were the main components of the fungus cell wall at all phosphoric acid concentrations and significant variations were observed in the concentrations of these components at different phosphoric acid levels.

**Figure 1:**
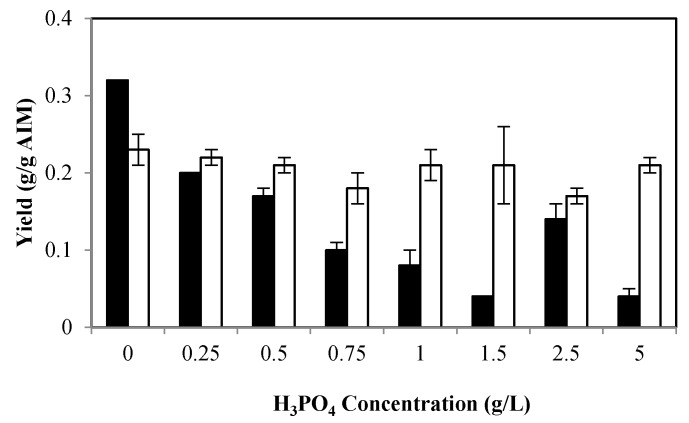
Effect of different phosphate concentration on glucosamine (GlcN) yield (g/g alkali-insoluble material (AIM)) (black bars) and *N*-acetyl glucosamine (GlcNAc) yield (g/g AIM) (white bars). Error bars represent the ± standard deviation (SD) of values obtained from independent experiments performed in triplicate. Average SD: *p =* 0.00 (black bars); *p* < 0.02 (white bars).

As shown in Figure 1, the highest glucosamine (GlcN) and *N*-acetyl glucosamine (GlcNAc) yields (0.32 and 0.23 g/g AIM, respectively) were obtained in the medium with no phosphoric acid. At 0.25 g/L H_3_PO_4_, a considerable decrease in GlcN yield (62%) was observed. The yield continually as decreased at higher phosphoric acid levels such that it dropped to 0.04 g/g AIM at 1.5 g/L phosphoric acid. Unexpectedly, the GlcN concentration increased to 0.14 g/g AIM at 2.5 g/L phosphoric acid, while it decreased to 0.04 g/g AIM at 5 g/L phosphoric acid. GlcN is a major structural component of chitosan and, therefore, the solution without H_3_PO_4_ was the most suitable culture medium for chitosan production. Although increasing the phosphoric acid concentration was generally accompanied by a reduction in GlcNAc content, a lower variation in the yield of this component was observed compared to that of GlcN (Figure 1).

The sum of GlcN and GlcNAc, as a representative of the total chitin and chitosan content of the cell wall, was highest in the absence of phosphate in the culture (55%). Furthermore, in general, a significantly lower chitin and chitosan content was achieved at higher phosphoric acid levels (Table 1).

As indicated in Table 1, the phosphate content of the cell wall was considerably lower for the fungus cultivated in the absence of phosphoric acid (0.047 g/g AIM). However, the concentration of phosphoric acid did not have a significant impact on the phosphate content of the cell wall (the average yield of phosphate was 0.129 g/g AIM in the presence of H_3_PO_4_).

### 2.2. The Effects of Potassium

#### 2.2.1. Effects of Potassium on Biomass Production and Its Composition

**Figure 2:**
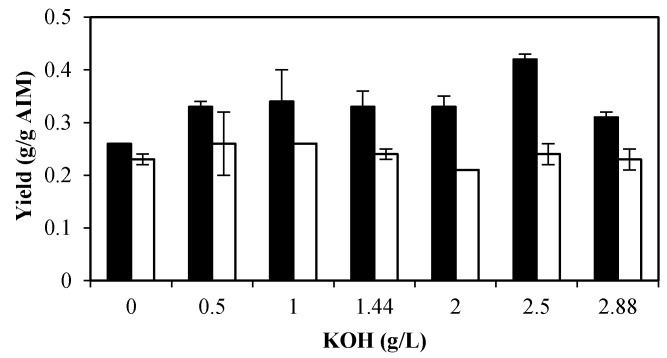
Effect of different potassium hydroxide concentrations on GlcN yield (g/g AIM) (black bars) and GlcNAc yield (g/g AIM) (white bars). Error bars represent the ± standard deviation (SD) of values obtained from independent experiments performed in triplicate. Average SD: *p* <0.02.

The highest chitin and chitosan levels were obtained in the medium with no phosphoric acid (Figure 1) and ethanol and glycerol yields were relatively high at this condition (Table 1). Therefore, the medium with no phosphoric acid was supplemented with different concentrations of KOH and changes in the yield of the products are summarized in Table 2 and Figure 2. The fungal growth was enhanced by increasing the potassium hydroxide concentration from 0.0 to 2.5 g/L (from 2.44 to 3.32 g/L, respectively). A slightly lower biomass concentration (3.10 g/L) was achieved at 2.88 g/L KOH. Potassium concentration had no significant effect on protein and AIM yields (Table 2).

#### 2.2.2. Effect of Potassium on Ethanol and Glycerol Production

According to Table 2, glycerol and ethanol yields were 0.06 g/g sugar and 0.44 g/g sugar, respectively, in the medium without KOH. No clear trend in ethanol and glycerol yields was observed at different concentrations of KOH. The minimum levels of ethanol and glycerol yields were obtained in the presence of 1 g/L of KOH (0.35 and 0.04 g/g sugar, respectively) (Table 2).

#### 2.2.3. Effects of Potassium on Cell Wall Composition

Generally, the presence of KOH improved the GlcN content of the cell wall. In the absence of KOH, the GlcN yield was 0.26 g/g AIM. This was enhanced to 0.33 g/g AIM by adding 0.5 g/L KOH. The highest GlcN content was 0.42 g/g AIM, which was achieved in the medium with 2.5 g/L KOH (Figure 2). Unlike GlcN, GlcNAc and phosphate yields were not considerably affected by the presence of KOH (Figure 2 and Table 2). The sum of chitin and chitosan was also enhanced in the presence of KOH and the maximum chitin and chitosan content was obtained at 2.5 g/L KOH (0.66 g/g AIM).

### 2.3. The Effects of Trace Metals

#### 2.3.1. Effects of Trace Metals on Biomass Production and Its Composition

At all KOH concentrations, biomass yield was enhanced by the addition of trace metals (8%–29%). The highest increase was observed in the medium containing no potassium (29%). By contrast, a 6%–14% reduction in protein content of biomass was observed in the presence of trace metals (Table 2).

#### 2.3.2. Effect of Trace Metals on Ethanol and Glycerol Production by *Mucor indicus*

As shown in Table 2, adding trace metals generally resulted in the reduction of ethanol and glycerol yields. By the addition of trace metals, in the absence of KOH, 14% and 17% reductions were observed in ethanol and glycerol yields, respectively. At different KOH concentrations, the addition of trace metals resulted in 7%–53% and 14%–17% reductions in ethanol and glycerol yields, respectively.

#### 2.3.3. Effects of Trace Metals on Cell Wall Composition

**Figure 3:**
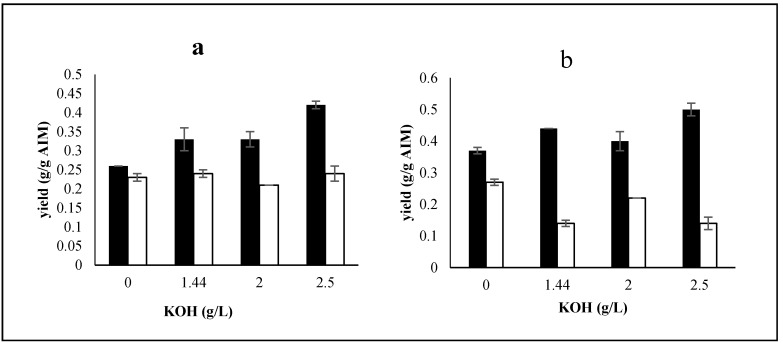
Effect of trace metals at different levels of KOH (without H_3_PO_4_) on GlcN (g/g AIM) (black bars) and GlcNAc yields (g/g AIM) (white bars): (**a**) cultures without trace metals; (**b**) cultures including trace metals. Error bars represent the ± standard deviation (SD) of values obtained from independent experiments performed in triplicate. Average SD: *p* < 0.015.

A 26% increase was observed in AIM yield by adding trace metals to culture containing 2.5 g/L potassium hydroxide. However, trace metals did affect the AIM yield at other KOH concentrations (Table 2). Adding a trace metals solution to the culture medium increased glucosamine in the fungal cell wall. In the presence of trace metals, GlcN yield was increased by 42%, 33%, 21%, and 21% at KOH concentrations of 0, 1.44, 2, and 2.5 g/L, respectively (Figure 3). According to Figure 3, there was no obvious trend in the changes of GlcNAc content. Furthermore, no significant effect on phosphate yield was observed. The total chitosan and chitin of the cell wall were remarkably higher in the presence of trace metals (58–64 g/g AIM) (Table 2).

### 2.4. The Effects of Yeast Extract

#### 2.4.1. Effects of Yeast Extract on Biomass Production and Its Composition

Several media with different concentrations of yeast extract were prepared to investigate the effects of yeast extract. The optimum concentration of phosphates and potassium obtained in previous experiments for chitosan production was also applied. Moreover, trace metals were added to all samples because of their positive effects on chitosan production. No fungal biomass was formed in the absence of yeast extract. Moreover, 0–3.16 g/L biomass was produced in the presence of 0–7 g/L yeast extract. The highest biomass yield was obtained at 7 g/L yeast extract, which was 93% higher than the yield obtained at 1 g/L yeast extract (Table 3).The protein content of the biomass was 48%–59%. The minimum protein content belonged to the biomass grown in the presence of 1 g/L yeast extract.

#### 2.4.2. Effect of Yeast Extract on Ethanol and Glycerol Production by *M. indicus*

Yeast extract showed a significantly positive effect on ethanol yield. A very low ethanol yield (0.01 g/g sugars) was obtained in the presence of 1 g/L of yeast extract. Doubling the amount of yeast extract resulted in an enhancement of ethanol yield to 0.12 g/g sugar. The highest ethanol yield was 0.43 g/g sugars, which was obtained at 7 g/L yeast extract.

The lowest amount of yeast extract (1 g/L) resulted in a minimum yield of not only ethanol but also glycerol. Glycerol production was doubled by doubling the yeast extract concentration. According to Table 3, maximum yields of glycerol were observed at concentrations of 6 and 7 g/L yeast extract. At these concentrations, glycerol production was four times greater than that in the media with 1 g/L of yeast extract.

#### 2.4.3. Effects of Yeast Extract on Cell Wall Composition

**Figure 4:**
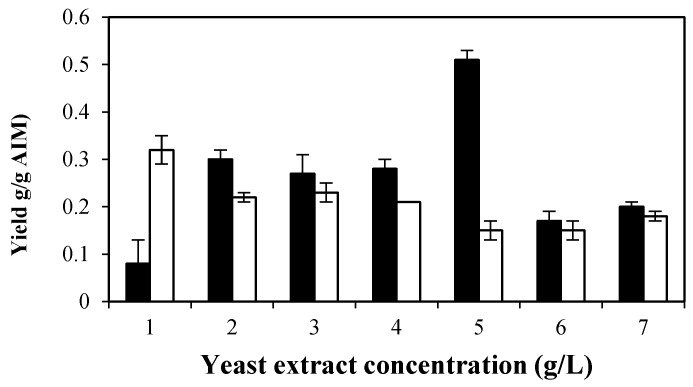
Effect of different concentrations of yeast extract on GlcN yield (g/g AIM) (black bars) and GlcNAc yield (white bars) (g/g AIM). Error bars represent the ± standard deviation (SD) of values obtained from independent experiments performed in triplicate. Average SD: *p* <0.03 (black bars); *p* <0.015 (white bars).

As shown in Table 3, by increasing the biomass production in the presence of yeast extract, the AIM yield was reduced. Maximum AIM yield (0.22 g/g biomass) was obtained at 1 g/L of yeast extract and was 57% more than the minimum yield (0.14 g/g biomass), which occurred at 6 g/L yeast extract. As illustrated in Figure 4, an enhancement in GlcN yield (from 0.08 to 0.51 g/g AIM) was observed by increasing the concentrations of yeast extract (up to 5 g/L). However, GlcN yield was reduced at higher concentrations of yeast extract.

Additionally, yeast extract significantly affected the GlcNAc yield (Figure 4). The highest GlcNAc yield (0.32 g/g AIM) was obtained in the presence of 1 g/L of yeast extract. Higher concentrations of yeast extract, however, reduced the GlcNAc. The highest total chitin and chitosan were obtained at 5 g/L of yeast extract. Accordingly, a yeast extract concentration of 5 g/L was selected as the optimal concentration for enhancing chitosan production.

In this study, phosphate yield followed an inverse trend to that of total chitosan and chitin yield. The highest phosphate content of the cell wall (0.16 g/g AIM) was obtained at 6 g/L yeast extract, when the total chitosan and chitin were at a minimum (0.32 g/g AIM).

## 3. Discussion

Economic aspects seems to be the bottleneck of fungal chitosan production in commercial scales. This is because fungal chitosan has lower yields compared to chitosan obtained from shellfish waste. However, taking into account the superior properties of fungal chitosan, it is worth enhancing the yield in order to improve the economy of the process. The purpose of the current study was to investigate the effect of a formulation of fungal culture media on chitosan yield obtained by *M. indicus.*

Chitosan, chitin, and polyphosphates are the main ingredients of the cell wall of zygomycetes fungi [13]. Bartnicki-Garcia and Nickerson [14] reported 23.3% and 22.1% phosphate in the cell wall of filamentous and yeast forms of *M. indicus*, respectively. A phosphate content of 8.3%–10% was reported in the cell wall of a filamentous form of *M. indicus* by Jeihanipour et al. [15]. Naghdi et al. [16] reported the possibility of the presence of a chitosan–phosphate salt in the cell wall of zygomycetes. In the current study, removal of phosphoric acid from the culture medium was accompanied by tge maximum production of chitosan. It should be mentioned that under this condition the medium contained 0.14 g/L phosphate because of the presence of phosphate in yeast extract. According to Table 3, no fungal biomass was formed in the absence of yeast extract. Therefore, at this stage the effects of phosphoric acid were investigated at a constant yeast extract concentration (5 g/L).

Mohammadi et al. also showed that GlcN yield is increased at lower concentrations of potassium dihydrogen phosphate [17]. Similarly, the results of this study indicate that a maximum concentration of chitosan can be produced at lower amount of phosphoric acid. Accumulation and storage of different anionic materials, such as polyphosphates, in the cell wall of zygomycetes fungi, are suggested to be performed by chitosan, which is a cationic bioplymer [1]. Therefore, at very low concentrations of phosphates, chitosan synthesis is probably enhanced in order to increase the uptake of the phosphates from the culture. However, further investigations are needed in order to prove this theory.

Potassium is an important ion that affects the growth and survival of living cells [18]. The effect of potassium on the growth of *Rhizopus oligosporus* was reported by Peñaloza et al. using a computerized system, and a linear relationship between the biomass yield and K^+^ concentration (1–10 mg/L) in a liquid culture was confirmed [19]. Furthermore, it has been reported that higher chitosan production level can be obtained when fungal cells grow faster [1]. Enhancement of chitosan production in the presence of potassium in this study might be an example of this phenomenon.

Some metals at very low concentrations have fundamental roles in microbial growth and metabolism, while a higher dose of these metals may inhibit cell growth [20]. The effects of iron, zinc, boron, manganese, and molybdenum on the growth of *Lentinus cladopus Lév* were studied by Atri et al. and the maximum amount of fungal mycelium (7.46 mg/mL) was achieved at 1 ppm concentration of iron [21]. In addition, using trace metals to improve the biomass production and ethanol yield by *M. indicus* was investigated by Sues et al. [8]. Increasing ethanol and biomass yield by 60% and 73%, respectively, was achieved by adding 6.7 mL/L trace metals. In the current work, adding 1 mL/L trace metals to the culture medium showed a positive impact on the glucosamine yield. However, trace metals had a negative effect on ethanol and glycerol yield. Generally, an increase in the biomass yield and he chitosan concentration significantly reduces the ethanol yield. This general inverse relationship between ethanol and biomass yields in ethanolic fermentation has been discussed in previous studies [1,12].

Yeast extract contains many nutritional compounds, including various vitamins, minerals, amino acids, peptides, nucleic acids, and structural materials that are essential for the growth of microorganisms [1]. Sues [22] reported that the best performance of *M. indicus* was obtained by the addition of 5 g/L yeast extract to the medium. Similarly, in the current work, a sharp reduction was observed in all of the traits by decreasing the concentration of yeast extract. At a concentration of 5 g/L, optimum production of GlcN and GlcNAc was obtained and ethanol yield was satisfactory. Therefore, 5 g/L was chosen as the best concentration.

## 4. Materials and Methods

### 4.1. Microorganism

*M. indicus* CCUG 22424 was obtained from the Culture Collection at the University of Gothenburg (Göteborg, Sweden). The fungus was grown on agar plates containing (in g/L) 40 glucose, 20 agar, and 10 peptone at 32 °C for five days. The grown fungus was kept at 4 °C until use.

### 4.2. Cultivation of the Fungus 

A semi-synthetic solution [23] containing (in g/L), (NH_4_)_2_SO_4_ (7.5), MgSO_4_·7H_2_O (0.75), CaCl_2_·2H_2_O (1.0), and yeast extract (5) was used for cultivation. This was supplemented with different concentrations of H_3_PO_4_ and KOH (0–5 and 0–2.88 g/L, respectively). A trace metals solution was prepared according to Sues et al. [8] and added to the medium (1 mL/L).

The pH of solutions was adjusted to 5.5 prior to sterilization at 121 °C for 20 min. After cooling to room temperature, Indole-3-acetic acid (IAA) and kinetin (Kin) (1 mg/L of each) [12] were added to the solutions. Fungal spore suspension (2 × 10^5^ spores/mL) was used for inoculation (50 mL/L). The fungus was grown in 500 mL Erlenmeyer flasks with a 250 mL working volume for 48 h at 32 °C and 120 rpm. Afterwards, the fungal biomass was harvested by centrifugation at 4000× *g* for 10 min, washed three times with distilled water, freeze dried, and stored at room temperature for analysis.

### 4.3. Preparation of Trace Metals Solution

Ethylene diamine tetra acetic acid (6 g), CaCl_2_·2H_2_O (1.8 g), ZnSO_4_·7H_2_O (1.8 g), FeSO_4_·7H_2_O (1.2 g), H_3_BO_3_ (400 mg), MnCl_2_·4H_2_O (380 mg), Na_2_MoO_4_·2H_2_O (160 mg), CoCl_2_·2H_2_O (120 mg), CuSO_4_·5H_2_O (120 mg), and KI (40 mg) were dissolved in 1.5 L distilled water. Then, the pH was adjusted to 4.0 and the solution was diluted to 2 L. Finally, the solution was autoclaved at 120 °C for 20 min and kept at 4 °C until use [8].

### 4.4. Preparation of Alkali-Insoluble Material (AIM)

Freeze-dried biomass was treated with 30 mL of 2% sodium hydroxide solution at 120 °C for 20 min to produce alkali-insoluble material (AIM). Then, the mixture was centrifuged at 4000× *g* for 10 min and the obtained solid (AIM) was washed several times with distilled water to reach a neutral pH. The AIM was freeze-dried and kept at room temperature. This was analyzed as a representative for the fungus cell wall [23].

### 4.5. Determination of Glucosamine and N-Acetyl Glucosamine

The method reported by Mohammadi et al. [11] was employed to measure the concentrations of glucosamine (GlcN) and N-acetyl glucosamine (GlcNAc) in AIM. AIM (10 mg) was mixed with 0.3 mL of 72% (*v*/*v*) sulfuric acid for 90 min. Then, each sample was diluted by adding 8.4 mL of distilled water and heated at 121 °C for 1 h. The obtained AIM hydrolysates were reacted with NaNO_2_ to produce 2,5-anhydromannose. Ammonium sulfamate was used to neutralize the excess NaNO_2_. The obtained 2,5-anhydromannose and acetic acid were analyzed by High Performance Liquid Chromatography (HPLC, Jasco International Co., Tokyo, Japan) with an Aminex HPX-87H column (Bio-Rad, Richmond, CA, USA) equipped with a RI detector (Jasco International Co., Tokyo, Japan) using 0.6 mL/min eluent (5 mM sulfuric acid) at 60 °C. Finally, GlcN and GlcNAc yields were calculated using 2,5-anhydromannose and acetic acid concentrations [11].

### 4.6. Determination of Proteins and Phosphates

Determination of the protein contents of dried biomass was performed according to the Biuret method [24]. Ammonium molybdate spectrometric method (European standard ISO6878) was used for measuring the phosphate contents of the fungal cell wall (AIM) [25].

### 4.7. Analysis of Glucose, Ethanol, and Glycerol

Concentrations of glucose, ethanol, and glycerol were determined in the media, after 48 h of the fungal cultivation, using HPLC under similar conditions to those described for the analysis of acetic acid and 2,5-anhydromannose.

## 5. Conclusions

The concentration of different components in the culture media of *M. indicus* can significantly affect the fungal growth and chitosan and metabolite production. In this work, in a culture medium without phosphate, the presence of 2.5 g/L of KOH, 1 mL/L of trace metals and 5 g/L of yeast extract resulted in the highest chitosan yield.

## Figures and Tables

**Table 1:** Biomass, protein, alkali-insoluble material (AIM), phosphate yields, and sum of chitin and chitosan at different concentrations of H_3_PO_4_ (at 1.44 g/L of KOH).

H_3_PO_4_ (g/L)	Biomass ^a^ (g/L)	Protein ^b^ (g/g Biomass)	Glycerol ^c^ (g/g Sugar)	Ethanol ^c^ (g/g Sugar)	AIM ^b^ (g/g Biomass)	Phosphate ^b^ (g/g AIM)	Sum of Chitin and Chitosan ^d^ (g/g AIM)
0	3.36 ± 0.09	0.57 ± 0.00	0.06 ± 0.00	0.41 ± 0.01	0.21 ± 0.03	0.05 ± 0.00	0.55 ± 0.02
0.25	3.56 ± 0.13	0.57 ± 0.01	0.08 ± 0.00	0.46 ± 0.00	0.19 ± 0.02	0.11 ± 0.01	0.42 ± 0.01
0.5	3.62 ± 0.12	0.58 ± 0.01	0.08 ± 0.00	0.47 ± 0.01	0.18 ± 0.01	0.14 ± 0.02	0.38 ± 0.02
0.75	3.76 ± 0.10	0.60 ± 0.00	0.07 ± 0.00	0.44 ± 0.00	0.17 ± 0.01	0.15 ± 0.02	0.28 ± 0.03
1	3.78 ± 0.07	0.57 ± 0.04	0.09 ± 0.00	0.46 ± 0.00	0.17 ± 0.01	0.14 ± 0.00	0.29 ± 0.04
1.5	3.84 ± 0.13	0.55 ± 0.00	0.09 ± 0.00	0.46 ± 0.03	0.17 ±0.00	0.13± 0.01	0.25 ± 0.05
2.5	3.88 ± 0.05	0.59 ± 0.01	0.07 ± 0.00	0.46 ± 0.01	0.18 ± 0.00	0.13 ± 0.01	0.37 ± 0.03
5	4.11 ± 0.12	0.58 ± 0.02	0.07 ± 0.00	0.46 ± 0.01	0.17 ± 0.01	0.10 ± 0.00	0.24 ± 0.02

All experiments were performed in triplicate. Average standard deviation (SD): ^a^ *p* < 0.11; ^b^ *p* < 0.01; ^c^ *p* = 0.00; ^d^ *p* < 0.03.

**Table 2:** Biomass, protein, AIM, phosphate yields, and sum of chitin and chitosan in different concentrations of KOH (without H_3_PO_4_).

KOH (g/L)	Presence of Trace Metals	Biomass Yield ^a^ (g/L)	Protein Yield ^b^ (g/g Biomass)	Glycerol ^b^ (g/g Sugar)	Ethanol ^b^ (g/g Sugar)	AIM Yield ^b^ (g/g Biomass)	Phosphate Yield ^c^ (g/g AIM)	Sum of Chitin and Chitosan ^d^ (g/g AIM)
0.00	−	2.44 ± 0.08	0.6 ± 0.03	0.06 ± 0.00	0.44 ± 0.01	0.12 ± 0.02	0.005 ± 0.00	0.49 ± 0.01
–	+	3.12 ± 0.13	0.56 ± 0.03	0.05 ± 0.00	0.38 ± 0.00	0.14 ± 0.01	0.010 ± 0.00	0.64 ± 0.02
0.50	−	2.60 ± 0.06	0.59 ± 0.02	0.07 ± 0.00	0.39 ± 0.04	0.12 ± 0.00	0.020 ± 0.00	0.59 ± 0.04
1.00	−	2.60 ± 0.06	0.59 ± 0.00	0.04 ± 0.00	0.35 ± 0.01	0.13 ± 0.00	0.005 ± 0.00	0.60 ± 0.02
1.44	−	2.72 ± 0.09	0.60 ± 0.00	0.06 ± 0.00	0.47 ± 0.00	0.11 ± 0.01	0.006 ± 0.00	0.57 ± 0.04
–	+	3.08 ± 0.07	0.51 ± 0.00	0.05 ± 0.00	0.22 ± 0.00	0.12 ± 0.01	0.003 ± 0.00	0.58 ± 0.01
2.00	−	2.84 ± 0.07	0.62 ± 0.00	0.07 ± 0.00	0.44 ± 0.00	0.11 ± 0.01	0.010 ± 0.00	0.54 ± 0.02
–	+	3.08 ± 0.12	0.53 ± 0.00	0.06 ± 0.00	0.41 ± 0.00	0.12 ± 0.01	0.010 ± 0.00	0.62 ± 0.03
2.50	−	3.32 ± 0.08	0.54 ± 0.00	0.07 ± 0.00	0.35 ± 0.00	0.15 ± 0.00	0.010 ± 0.00	0.66 ± 0.03
–	+	3.63 ± 0.07	0.5 ± 0.01	0.06 ± 0.00	0.31 ± 0.00	0.19 ± 0.01	0.010 ± 0.00	0.64 ± 0.04
2.88	−	3.10 ± 0.13	0.57 ± 0.01	0.06 ± 0.00	0.37 ± 0.01	0.13 ± 0.00	0.005 ± 0.00	0.54 ± 0.03

All experiments were performed in triplicate. Average SD: ^a^ *p* < 0.09; ^b^ *p* < 0.01; ^c^ *p* = 0.00; ^d^ *p* < 0.03. “+” indicates the presence of trace metals and “−” shows the absence of trace metals in culture medium.

**Table 3:** Biomass, protein, AIM, phosphate yields, and sum of chitin and chitosan in different concentrations of yeast extract.

Yeast Extract (g/L)	Biomass Yield ^a^ (g/L)	Protein Yield ^b^ (g/g Biomass)	Glycerol ^c^ (g/g Sugar)	Ethanol ^c^ (g/g Sugar)	AIM Yield ^b^ (g/g Biomass)	Phosphate Yield ^d^ (g/g AIM)	Sum of Chitin and Chitosan ^e^ (g/g AIM)
0.00	<0.01	–	–	–	–	–	–
1.00	1.64 ± 0.08	0.48 ± 0.03	0.02 ± 0.00	0.01 ± 0.00	0.22 ± 0.02	0.1 ± 0.06	0.40 ± 0.08
2.00	2.00 ± 0.10	0.54 ± 0.00	0.04 ± 0.00	0.12 ± 0.00	0.21 ± 0.00	0.02 ± 0.00	0.52 ± 0.03
3.00	2.56 ± 0.07	0.56 ± 0.01	0.05 ± 0.00	0.32 ± 0.00	0.18 ± 0.01	0.05 ± 0.01	0.50 ± 0.06
4.00	2.68 ± 0.08	0.58 ± 0.00	0.04 ± 0.00	0.34 ± 0.00	0.18 ± 0.02	0.05 ± 0.00	0.49 ± 0.02
5.00	2.88 ± 0.08	0.57 ± 0.03	0.06 ± 0.00	0.41± 0.00	0.17 ± 0.01	0.05 ± 0.01	0.66 ± 0.04
6.00	3.08 ± 0.07	0.58 ± 0.00	0.08 ± 0.00	0.39 ± 0.00	0.14 ± 0.01	0.16 ± 0.00	0.32 ± 0.04
7.00	3.16 ± 0.05	0.59 ± 0.00	0.08 ± 0.00	0.43 ± 0.00	0.15 ± 0.00	0.11 ± 0.04	0.38 ± 0.02

All experiments were performed in triplicate. Average SD: ^a^ *p* < 0.09; ^b^ *p* < 0.01; ^c^ *p* = 0.00; ^d^ *p* < 0.02; ^e^ *p* < 0.04.
